# Comparison of distal transradial approach versus conventional transradial approach for coronary angiography and percutaneous coronary intervention: A prospective observational study

**DOI:** 10.1016/j.heliyon.2023.e17150

**Published:** 2023-06-14

**Authors:** Chunguang Feng, Bin Zong, Yi Liu, Mei Chen, Shanshan Li, Dujuan Xu, Bing Han

**Affiliations:** a Department of Cardiology, Xuzhou Central Hospital, Xuzhou, 221000, China; b Department of Ultrasound, Xuzhou Central Hospital, Xuzhou, 221000, China

**Keywords:** Snuffbox area, Radial artery, Distal radial artery, Coronary angiography, Percutaneous coronary intervention

## Abstract

**Background:**

Compared with the conventional transradial approach (TRA), there are limited data on the efficacy and safety of the novel distal transradial approach (DTRA). This study aimed to verify the effectiveness and safety of the DTRA for percutaneous coronary angiography and intervention. Besides, we also try to highlight the potential of the DTRA in reducing radial artery occlusion (RAO), shorter time to hemostasis, and improved patient comfort.

**Methods:**

This single-center prospective observational study enrolled patients treated with DTRA (n = 527) in the first 9 months and with TRA (n = 586) in the next 8 months from May 2020 to December 2021. The primary endpoint was the proximal RAO rate at 30 days.

**Results:**

Baseline data were similar between the two groups. The proximal radial artery occlusion rate at 30 days [2.3% vs. 7.0%], the success rate of puncture [86.4% vs. 96.7%], the Numeric Rating Scale score [1.97 ± 1.89 vs. 4.61 ± 2.68], and the incidence of postoperative subcutaneous hematoma and finger numbness [3.4% vs. 8.2%, 2.7% vs. 4.4%] were lower. The puncture time [6.93 ± 7.25 min vs. 3.18 ± 3.52 min] was longer, and the time until radial compression device removal was shorter [CAG: 138.61 ± 38.73 min vs. 191.6 ± 61.22 min, PCI:221.46 ± 62.45 min vs. 276.28 ± 76.39 min] in the DTRA group than TRA group (all P < 0.05). Multivariate logistic regression analysis revealed that the DTRA (OR 0.231, 95% confidence interval [CI] 0.088–0.769, P = 0.001),BMI<18.5 kg/m^2^ (OR 2.627, 95% CI 1.142–4.216, P = 0.004), Diabetes mellitus (OR 2.15, 95%CI1.212–3.475, P = 0.014), RCD removal time (CAG,min) (OR 1.091, 95% CI 1.013–1.441, P = 0.035) and RCD removal time (PCI,min) (OR 1.067, 95% CI 1.024–1.675, P = 0.022) were the independent risk factors of RAO 1 month after intervention procedure.

**Conclusion:**

DTRA was found to a lower incidence of postoperative RAO and bleeding-related complications, shorter time to achieve hemostasis, and greater patient comfort.

## 1 Introduction

Transradial approach (TRA) used for coronary angiography (CAG) and percutaneous coronary intervention (PCI) has been reported to have more advantages than the femoral approach, including fewer complications related to the approach site such as massive hemorrhage, subcutaneous hematoma, pseudoaneurysm, nerve injury, and similar. The shortening of time to achieve hemostasis can reduce the patient's bedtime and improve the comfort of early walking [1]. Therefore, as a standard method, TRA is recommended by current guidelines and consensus for performing CAG and PCI in stable angina pectoris and acute coronary syndrome [2,3]. However, TRA still has high incidences of postoperative hematoma, nerve injury, radial artery spasm, radial artery occlusion (RAO), and other complications, among which RAO is the most serious one. Once RAO occurs, the patient may experience a strange sensation in the hands, whose function may also be affected. Furthermore, this may also affect TRA hemodialysis treatment of arteriovenous fistula. Moreover, coronary intervention cannot be performed via TRA again, and coronary artery bypass grafting cannot be performed as a bridge vessel [4,5]. A previous study showed that the incidence of RAO in the first 24 h after TRA was 7.5%, which was reduced to 5.5% in the 30 days after TRA [6].

A novel technique of the distal transradial approach (DTRA) in the anatomical snuffbox (AS) area was first described by Kiemeneij et al. [7]. Its advantage is that the puncture is located at the distal end of the superficial palmar arch bifurcation, which can retain anterograde blood flow to reduce the risk of hand ischemia; and the incidence of RAO is also low. Considering anatomy and function which the puncture site of DTRA is superficial and the bone structure is below it, the hemostasis was faster as well as more comfortable. As a result, there was less bleeding and neurological complications. Therefore, Sgueglia et al. [8] considered that DTRA was a very promising and alternative intervention path. As the position of the blood vessel at the puncture site is shallow and the bone structure is under it, the hemostasis occurs faster. In recent years, increasing studies have focused on the feasibility and safety of performing coronary procedures via DTRA [9]. Nevertheless, studies on the puncture success rate of different populations, the incidence of RAO, and the impact on patients’ comfort are still lacking.

The main purpose of our study was to compare the incidence of RAO after CAG and PCI through DTRA and TRA by Doppler ultrasound in the real world. Moreover, hemostasis, complications, and other aspects of the two approaches were compared.

## 2 Methods

### 2.1 Study design

This prospective observational study of the safety and efficacy of DTRA vs. TRA included patients undergoing CAG and PCI at the Department of Cardiology in our hospital, over a 17 months period between May 2020 and October 2021. Inclusion criteria were the following: patients who underwent CAG and PCI for the first time; the all-comers method was used. The right radial artery or distal radial artery was selected, and a 6F sheath was used. DTRA was performed in the patients treated during the first 9 calendar months. After failure, patient was crossovered to TRA completed the operation. During the following 8 calendar months, TRA was directly performed in patients used as the control group. After TRA failed, patient was crossovered to alternate access, such as brachial artery or ulnar artery.

Exclusion criteria:The reasons from enrollment: (1) If Allen test (+), the obstruction of the ulnar artery was considered; (2) Radial artery or distal radial artery didn't be touched a palpable pulse; (3) The hemodynamic indicators were unstable; (4) The patient refused to sign informed consent. The reasons from the analysis: (1) Case who did not complete 30-day follow up; (2) Case who was acute myocardial infarction undergoing direct PCI, it may take too long to puncture the distal radial artery, which may affect the time to reach reperfusion. The operation was completed directly through TRA. (3) Case who was crossover to alternate access after DTRA, as puncture failure may cause local injury due to multiple punctures of the distal radial artery, thus affecting the comparison between groups; (4) Case who was the failed puncture of the radial artery in the TRA group.

Basic information of the patients was recorded at admission, including age, gender, height and weight, cardiovascular risk factors, cardiac and renal function, related drug use, type of operation, etc. The patients were followed up 30 ± 3 days after the operation to evaluate their clinical conditions and ultrasonography.

This work has been carried out in accordance with the Declaration of Helsinki (1975) of the World Medical Association. This study was approved by the institutional ethics committee of our hospital, and all participants provided written informed consent.

### 2.2 Color Doppler ultrasound imaging

All patients underwent ultrasound examination of the radial artery 1 day before operation and 30 days after operation [10]. The color Doppler ultrasound diagnostic instrument PHILIPS EPIQ 7C (Koninklijke, Philips N.V., the Netherlands) and linear array probe L12-3 were used to measure the diameter, cross-sectional area, and peak velocity of the radial artery and distal radial artery. Color Doppler ultrasound showed no obvious blood flow signal in the lumen of the right radial artery, which was defined as RAO. Distal RAO (DRAO) was defined as flow reversal on color Doppler ultrasound around the radial artery in the nasal snuffbox area. lumen inner diameter, cross sectional area and peak velocity was measured. All data were measured three times, and the average value was taken. All cases were examined and interpreted by two experienced doctors.

### 2.3 Puncture and hemostasis procedure

The patient's snuff bottle area was palpated before the operation. The part with the obvious pulsation of the distal radial artery in the snuff bottle area was selected for DTRA. “Anatomical snuffbox” refers to a concave area of a triangle formed by abductor pollicis longus tendon, extensor pollicis longus tendon, extensor pollicis brevis tendon, and radial stem. In the snuff pot area, the pulse of the distal radial artery can be palpated. Local anesthesia was performed by injecting 0.5 ml lidocaine. Seldinger's puncture method was used; the 20G puncture trocar (Terumo Corp., Tokyo, Japan) was punctured from the outside to the inside 30–45° towards the traditional (wrist) radial artery. After a successful puncture, the 0.021″ hydrophilic coating guide wire was pushed into the artery as the guide track, and the 6F radiocus integrator II was sent into the (Terumo Corp., Tokyo, Japan) sheath. Routine 500 μg nitroglycerin was injected along the arterial sheath. Heparin 5000U was used in CAG. PCI required the use of additional heparin (120u/kg) in the arterial sheath according to the patient's weight to maintain the activated clotting time (ACT) at 250–300 s. After the operation, the radial artery sheath was removed and wrapped with a gauze gasket and elastic tape. If the distal radial artery was not successfully punctured five times, it was identified as puncture failure, and the operation was completed by other paths (These cases were successfully completed by TRA).

In the control group, the point with the strongest radial artery pulse 2–3 cm below the palmar transverse stria of the right radial artery was selected as the puncture point. The puncture trocar, sheath, and drugs used were the same as those in the DTRA group. TR Band™ radial artery hemostat (Terumo Corp., Tokyo, Japan) was used to compress and stop bleeding after the operation.

The two groups were decompressed 1.5h after CAG or 3h after PCI via DTRA and 2.5h after CAG or 3.5h after PCI via TRA to check for bleeding. If there was no bleeding, the compression material was completely removed. The gauze was pressed and fixed again, and the air bag of the compressor was inflated and pressurized if there was bleeding. Then, they were checked every 10min until there was no bleeding, and the compression material was removed.

The success rate of puncture, the time of arterial puncture (from the first local infiltration anesthesia to the time after the insertion of the arterial sheath), and the time before radial compression device (RCD) removal were recorded.

The whole study process was completed by an experienced operator (who had 15 years of experience in PCI with more than 2000 cases via TRA and 200 cases via DTRA).

### 2.4 Outcomes

The primary endpoint was the radial artery occlusion (RAO), which was defined as slow or no blood flow on two-dimensional color Doppler, or low-velocity signal on pulse Doppler.

Secondary endpoints were: (1) ultrasonic indexes (RAO,DRAO, Lumen inner diameter, Cross sectional area and Peak velocity) of the distal radial artery and proximal radial artery before and 30 days after operation; (2) puncture success rate was defined as the proportion of sheath successfully inserted into the radial artery; (3) arterial puncture time was defined as the time from the first contact of the puncture needle with the skin to the insertion of the sheath; (4) numerical rating scale (NRS) [11]: the scale of 0–10 was used to mark the pain intensity levels of different degrees according to patient's perception, where 0 indicated no pain, 10 indicated the most intense pain, <4 indicated mild pain (does not affect sleep), 4–7 indicated moderate pain, and >7 indicated severe pain (resulting in an inability to sleep or waking up from sleep). (5) Time until radial compression device (RCD) removal was defined as the time when the sheath was removed immediately after the operation until the compressor (object) was completely removed; (6) Complications included bleeding such as subcutaneous hematoma ≧5 cm in diameter including forearm hematoma and hematoma limited to hand, arteriovenous fistula, pseudoaneurysm, osteofascial compartment syndrome, nerve injury (motor or sensory nerve injuries, such as numbness and paresthesia).

### 2.5 Statistical analysis

SPSS 22.0 statistical software (IBM, Armonk, New York) was used to perform statistical analysis. The measurement data were described as mean ± standard deviation. The counting data were expressed as a proportion (%). Student's t-test and the Kruskal–Wallis test were used to test the differences between measurement data groups, while the Chi-squared test or Fisher's exact test was used to compare counting data. Multivariate logistic regression analysis was used to explore the possible factors associated with the incidence of RAO in 1 month after intervention procedure. P < 0.05 indicated statistical significance.

## 3 Results

**Fig. 1:**
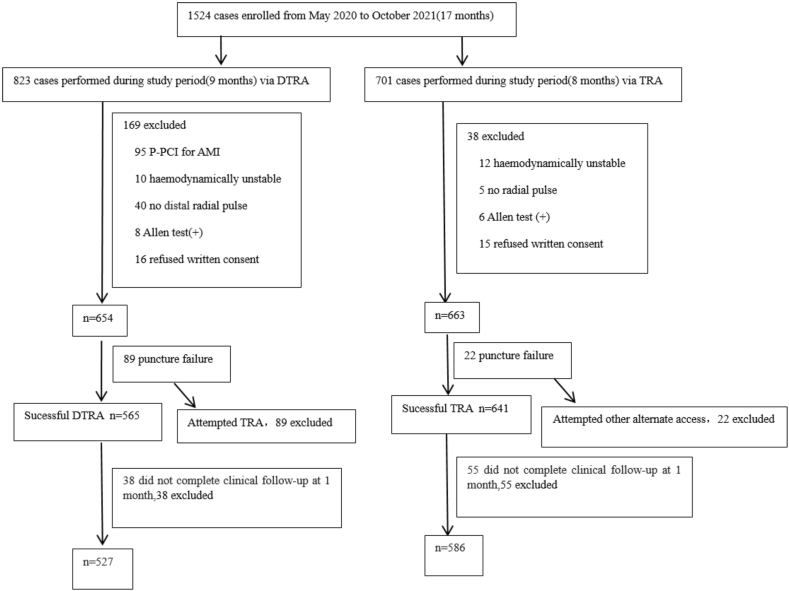
Study flow diagram. A prospective observational study in patients undergoing CAG/PCI via DTRA vs. TRA.

According to the procedure in Fig. 1, the patients who underwent CAG/PCI in our department from May 2020 to October 2021 were included in the study. Among them, 527 patients underwent DTRA in the first 9 months and 586 patients underwent TRA (control group) in the next 8 months,. There was no significant difference in the characteristics of basic data between the two groups (P > 0.05, Table 1).

**Table 1:** Baseline clinical characteristics.

Baseline Characteristics	DTRA Group (n = 527)	TRA Group (n = 586)	*P*-value
Age, y	65.8 ± 16.7	66.2 ± 18.2	0.36
Females/Male	138/389 (26.2%/73.8%)	163/423 (27.8%/72.2%)	0.54
Weight (kg)	63.4 ± 11.6	62.8 ± 11.2	0.82
Height (cm)	161.3 ± 9.5	161.7 ± 8.9	0.61
BMI	24.2 ± 6.8	24.7 ± 6.1	0.71
**Cardiovascular risk factors**			
Hypertension, n (%)	427 (81%)	486 (83%)	0.41
Diabetes Mellitus, n (%)	206 (39.1%)	214 (36.5%)	0.38
Dyslipidemia, n (%)	202 (38.4%)	253 (43.1%)	0.101
smoking history	315 (59.8%)	364 (62.1%)	0.42
Prior myocardial infarction, n (%)	47 (8.9%)	50 (8.6%)	0.82
Prior stroke, n (%)	40 (7.6%)	42 (7.1%)	0.79
Heart Failure with EF <40%	65 (12.4%)	69 (11.8%)	0.77
Creatinine clearance (mL/min) < 50%	41 (7.8%)	52 (8.9%)	0.51
**Indications for CAG/PCI**			
Acute Coronary Syndromes (ACS)	143 (27.1%)	156 (26.6%)	0.85
Chronic Coronary Syndromes (CCS)	157 (29.8%)	189 (32.3%)	0.38
Others	227 (43.1%)	241 (41.1%)	0.51
**Medications (30 days after CAG/PCI)**			
Dual antiplatelet therapy, n (%)	149 (28.2)	172 (29.4)	0.69
Aspirin, n (%)	244 (46.3)	251 (42.8)	0.25
Ticagrelor, n (%)	140 (26.5)	161 (27.4)	0.73
Clopidogrel, n (%)	9 (1.7)	12 (2.0)	0.68
Dual antiplatelet plus anticoagulant therapy, n (%)	44 (8.3)	56 (9.6)	0.48
Warfarin, n (%)	39 (7.4)	48 (8.2)	0.62
Direct oral anticoagulant, n (%)	5 (1.0)	8 (1.4)	0.52
**Procedure Type**			
CAG, n (%)	339 (64.4)	389 (66.3)	0.47
PCI, n (%)	188 (35.6)	197 (33.7)	0.47

### 3.1 Primary endpoint

Color Doppler ultrasound examination 30 days after operation revealed that 12 cases in the DTRA group had RAO, which was located in the forearm segment of the radial artery, and 41 cases in the TRA group had RAO, which was located in the puncture point of the radial artery and the forearm segment of the radial artery. There was a significant difference between the two groups (P < 0.05). Also, there were 17 cases of DRAO in the DTRA group, all located at the puncture point of the distal radial artery, and 10 cases of DRAO in the TRA group There was no significant difference between the two groups.

As shown in Table 2, univariate analysis revealed that DTRA,age>70 years, Diabetes mellitus,BMI<18.5 kg/m^2^ (Definitions.BMI = weight/height^2^, and BMI<18.5 kg/m^2^ was defined as low BMI [12]), heart failure with EF < 40%, RCD removal time (CAG,min) and RCD removal time (PCI,min) were potential risk factors associated with RAO 1 month after intervention (Table 2). Multivariate logistic regression analysis was further performed to evaluate the independent risk factors. It was found that the DTRA (OR 0.231, 95% confidence interval [CI] 0.088–0.769, P = 0.001) was independently negatively associated with RAO after adjusting for the other factors. BMI<18.5 kg/m2 (OR 2.627, 95% CI 1.142–4.216, P = 0.004), Diabetes mellitus (OR 2.08, 95% CI 1.117–3.658, P = 0.017), RCD removal time (CAG,min) (OR 1.091, 95% CI 1.013–1.441, P = 0.035) and RCD removal time (PCI,min) (OR 1.067, 95% CI 1.024–1.675, P = 0.022) were found independently positively associated with RAO after adjusting for the other factors (Table 3).

**Table 2:** Univariate analysis for radial artery occlusion.

Variables	RAO Yes (n = 53,%)	RAO No (n = 1060,%)	*P*-Value
Age>70 years (n = 167)	15 (28.30%)	152 (14.34%)	0.005
Females gender (n = 301)	18 (33.96%)	283 (26.70%)	0.245
BMI<18.5 kg/m2 (n = 38)	13 (24.53%)	25 (2.36%)	0.000
Hypertension (n = 913)	41 (77.36%)	872 (82.26%)	0.364
Diabetes mellitus (n = 420)	28 (52.83%)	392 (36.98%)	0.020
LDL-C>2.60mmol/Ｌ (n = 455)	24 (45.28%)	431 (40.66%)	0.504
Smoking history (n = 679)	37 (69.81%)	642 (60.57%)	0.178
Heart Failure with EF <40% (n = 134)	12 (22.64%)	122 (11.51%)	0.015
Creatinine clearance (mL/min) < 50% (n = 93)	6 (11.32%)	87 (8.21%)	0.424
ACS (n = 299)	16 (30.19%)	283 (26.70%)	0.576
Oral anticoagulant (n = 100)	3 (5.66%)	97 (9.15%)	0.386
PCI, n (%) (n = 385)	19 (35.85%)	366 (34.53%)	0.844
Baseline radial artery diameter<2.0 mm (n = 41)	4 (7.58%)	37 (3.49%)	0.126
RCD removal time (CAG,min)	183.22 ± 57.67	161.38 ± 42.69	0.019
RCD removal time (PCI,min)	272.57 ± 73.47	248.33 ± 65.21	0.021
DTRA (n = 527)	12 (22.64%)	515 (48.58%)	0.000

BMI: Body mass index; ACS: Acute Coronary Syndromes; RCD: radial compression device; DTRA: distal transradial approach.

**Table 3:** Predictors of radial artery occlusion by multivariate logistic analysis.

Variables	Odds ratio	95% CI	*P*-Value
Age>70 years	1.183	0.654–2.211	0.412
Diabetes mellitus	2.08	1.117–3.658	0.017
BMI<18.5 kg/m2	2.627	1.142–4.216	0.004
Heart Failure with EF < 40%	1.489	0.746–2.318	0.224
RCD removal time (CAG,min)	1.091	1.013–1.441	0.035
RCD removal time (PCI,min)	1.067	1.024–1.675	0.022
DTRA	0.231	0.088–0.769	0.001

BMI: Body mass index; DTRA: distal transradial approach; CI: confidence interval.

Meanwhile, the lumen diameter and cross-sectional area of the distal radial artery were smaller less compared with the proximal radial artery, the peak velocity was higher compared with the proximal radial artery (P < 0.05). Moreover, compared with before the operation, the lumen diameter and cross-sectional area were significantly increased, and the peak flow velocity was significantly decreased after the operation (P < 0.05, Table 4).

**Table 4:** Analysis of ultrasonic indexes of the distal radial artery and proximal radial artery.

	DTRA Group (n = 527)			TRA Group (n = 586)		
	1 day before operation	30 days after operation	P value	1 day before operation	30 days after operation	*P*-value
RAO, n (%)	0	12 (2.3%)^a^		0	41 (7.0%)	
DRAO, n (%)	0	17 (3.2%)^a^		0	10 (1.7%)	
Lumen inner diameter (mm)	2.05 ± 0.41^a^	2.10 ± 0.39^ab^	<0.05	2.59 ± 0.47	2.65 ± 0.40^b^	<0.05
Cross sectional area (mm2)	3.55 ± 1.70^a^	3.85 ± 1.50^ab^	<0.05	5.68 ± 2.06	6.51 ± 1.98^b^	<0.05
Peak velocity (cm/s)	68.89 ± 22.06^a^	63.53 ± 20.28^ab^	<0.05	56.21 ± 18.34	51.11 ± 18.62^b^	<0.05

The measurement data were described as mean ± SD. The counting data were expressed as a proportion (%).

RAO: radial artery occlusion; DRAO: distal radial artery occlusion.

RAO was defined as Color Doppler ultrasound showed no obvious blood flow signal in the lumen of the radial artery.

Distal RAO (DRAO)was defined as flow reversal on color Doppler ultrasound around the radial artery in the nasal snuffbox area.

^a^ P = 0.0002 for RAO at 30 days and ^a^ P = 0.10 for DRAO at 30 days after operation in the DTRA group and TRA group. ^a^ P < 0.05 for other indexes in the same period. The postoperative and preoperative ratio B of each index was ^b^P < 0.05.

### 3.2 Secondary endpoints

For the comparison of between the two groups, The puncture success rate in the DTRA group was significantly lower than that of the TRA group. The puncture time in the DTRA group was significantly longer than that in the TRA group, and the degree of postoperative pain in the DTRA group was significantly better than that in the TRA group, the RCD removal time (whether CAG or PCI) in the DTRA group was significantly lower than that in the TRA group, the subcutaneous hematoma and finger numbness in the DTRA group was significantly better than that in the TRA group. The arteriovenous fistula was rare, while there were no other complications (Table 5).

**Table 5:** Procedural and post-procedural characteristics.

	DTRA Group (n = 527)	TRA Group (n = 586)	*P*-value
Success rate of puncture (%)	86.40%	96.70%	<0.05
Puncture time (min)	6.93 ± 7.25	3.18 ± 3.52	<0.05
NRS	1.97 ± 1.89	4.61 ± 2.68	<0.05
RCD removal time (CAG, min)	138.61 ± 38.73	191.6 ± 61.22	<0.05
RCD removal time (PCI, min)	221.46 ± 62.45	276.28 ± 76.39	<0.05
Subcutaneous hematoma, n (%)	18 (3.4)	48 (8.2)	<0.05
Numbness in the fingers, n (%)	14 (2.7)	26 (4.4)	<0.05
AV fistula	1	1	

RCD: radial compression device, NRS: numerical rating scale, AV fistula: arteriovenous fistula.

## 4 Discussion

The results showed that DTRA had a high success rate, low postoperative RAO rate, with few bleeding-related complications. DTRA can be used as an effective supplement to the coronary procedure approach. DTRA should be given priority if the patients need multiple PCI diagnoses and treatments, bilateral coronary angiography, or other treatment such as arteriovenous fistula surgery.

An important complication of TRA is RAO. Kotowycz et al. revealed that the incidence of RAO ranged from 1% to 10% [13]. A systematic review reported that incidence of RAO was 7.5% in the first 24 h after the operation, and it could decrease to 5.5% in the subsequent 30 days of follow-up [6]. RAO is usually asymptomatic and does not tend to cause serious complications since a dual blood supply protects the hand. However, once the RAO occurs, this artery cannot be reused for future intervention procedure, hemodialysis arteriovenous fistula preparation, or coronary artery bypass grafting (CABG) [14]. Some studies found that RAO might be related to some factors such as the patient's own clinical characteristics (including age, gender, diabetes, radial artery diameter, etc.), the size of the sheath, puncture technology, the use of vasodilators, anticoagulation and hemostasis schemes, and the degree of arterial wall tissue damage at the puncture site and sheath expansion site [15,16]. Some measures, such as “patent hemostasis”, higher dose anticoagulant application, and shorter postoperative compression time, have been used to reduce the probability of RAO [17]. In addition, certain researchers have stopped bleeding in the radial artery and prevented ulnar compression by making the blood flow preferentially flow to the radial artery to further reduce the incidence of early RAO [18,19]. However, the rate of RAO is still high.

Kiemeneij et al. [7] firstly reported the effect of DRTA in reducing RAO, bleeding, and other complications. Moreover, Coughlan et al. [20] pointed out that if the patient was treated with left-handed interventional therapy, DRTA provided a better ergonomic position for the operator. When the patient's left hand is placed on the abdomen, the operator can operate on the right side of the patient as usual, and the radiation to the operator can be reduced. Sgueglia et al. [8] suggested that DTRA was a very promising and alternative intervention path from the perspective of anatomy and function. By DTRA, the puncture point is located between the branch supplying the superficial palmar arch to the proximal end and the short periosteal artery to the distal end. Even after the puncture site is blocked, it can maintain the downstream through the superficial palmar arch, thus reducing the risk of retrograde thrombosis in the radial artery of the forearm. Furthermore, it has been reported [21] that the puncture site has a shallow blood vessel surface and small vessel diameter, under which the bone structure lies. Therefore, the patient is more comfortable and the complications such as bleeding and nerves occur less frequently when the hemostasis is faster. In their single-center, prospective, randomized controlled trial that enrolled 282 patients undergoing transradial coronary procedure, Eid-Lidt et al. divided patients into the DTRA group (n = 140) and TRA group (n = 142). The condition of proximal RAO was evaluated by ultrasonography 24 h and 30 days after the operation. The RAO of 24 h after operation was 8.4% in the TRA group per protocol and 8.8% in an intention-to-treat analysis versus only 0.7% (P = 0.002) and 1.2% (P = 0.003) in the DTRA group, respectively. The RAO of 30 days after operation was 5.6% versus 0.7% (P = 0.019) per-protocol and 6.4% versus 0.7% (P = 0.007) in an intention-to-treat analysis. DTRA significantly reduced the occurrence of proximal RAO [22]. Another randomized controlled study had once again proved the advantages of DTRA [23]. Therefore, DTRA was also recommended by consensus for the first time [24].

In this observational study, both groups were treated with 6F sheaths. The baseline data of the two groups were balanced due to the relatively large enrollment. After one month, RAO in the DRTA group was significantly less frequent than that in the TRA group. However, there was no significant difference in DRAO between the two groups. DRAO and RAO could exist together or exist separately. DRAO was defined as flow reversal on color Doppler ultrasound around the radial artery in the nasal snuffbox area has been described separately in other study [24]. The reason for RAO in the DTRA group was that the sheath was long, and patients' blood vessels were thin. The sheath caused damage to local tissues when passing through the radial artery. The multivariate logistic regression analyses from this study suggested that the DTRA was an effective way to reduce RAO, while Patients with BMI<18.5 kg/m2, Diabetes mellitus, extended RCD removal time (CAG or PCI,min) were at high risk of developing RAO after intervention. The lumen diameter and cross-sectional area of the distal radial artery and proximal radial artery after operation increased compared with those before the operation. The peak flow velocity accordingly decreased, which may be related to the expansion of the sheath without local tissue damage, which is consistent with the research results of Mizuguchi et al. [25]. This study showed that the long puncture time and success rate of the DTRA group were significantly lower than those of the control group (6.93 ± 7.25 min vs. 3.18 ± 3.52 min; 86.4% vs. 96.7%, all P < 0.05). The success rate of this study was also lower compared with other related studies [25,26], which is probably because only patients without arterial pulsation were excluded. Patients were selected based on the all-comers principle as long as it was possible to feel the pulsation. In a previous study [27] by this study's team, the results showed that patients with diabetes, elderly female gender and thin lumen (<1.8–2.0 mm) of distal radial artery had a significant puncture failure rate. These patients were suggested to be chosen the radial artery route first to avoid the injury of the distal radial artery caused by multiple punctures.

The puncture failure reasons in this study included the puncture needle failed to be penetrated the arterial vessel (n = 36), the guide wire failed to enter arterial vessel (n = 49), and the sheath failed to be inserted smoothly (n = 4). The entering failure of the guide wire was the main manifestation, which was related to the guide wire entering the ulnar side of the deep palmar arch, the natural curvature of the distal radial artery from the palmar side to the dorsal side of the hand, and the tortuosity of the radial artery. Regardless of CAG or PCI, the RCD removal time in the DTRA group was significantly shorter than that in the TRA group. An index for evaluating postoperative pain, Numerical Rating Scale (NRS) [11], was also significantly lower compared to the TRA group, thus indicating that the patients in the DTRA group had fast postoperative hemostasis and significantly improved comfort. Postoperative complications between the two groups were only subcutaneous hematoma and numbness in the fingers. DTRA group was significantly better than the control group, and AV fistula was rare between the two groups.

The present study has the following limitations: (1) This was a single center observational study. The control group was prone to selective bias, and was not matched. Although this study used the same operator and a relatively large number of patients which ensured that there was no clinically significant difference in baseline indexes between the two groups, there was only temporal criterion (first 9 months vs later 8 months) for the randomization to each method. (2) A total of 93 patients did not complete the 1-month follow-up, and 111 patients were excluded due to puncture failure of two groups, which could lead to a selection bias. (3) 6F sheath was used in all patients. Thus, this study cannot confirm the feasibility and safety of larger-size sheaths. (4) The follow-up time was short. Compared with conventional radial artery puncture, large-scale randomized controlled clinical studies are needed to further verify the advantages and disadvantages of the proposed approach.

In conclusion, DTRA provides another puncture site choice for operators and has a high surgical success rate. Compared with TRA, the postoperative RAO rate is lower, and the incidence of bleeding-related complications is lower. Therefore, Through DTRA for CAG or PCI may be safe and feasible.

## Production notes

### Author contribution statement

Chunguang Feng: Conceived and designed the experiments; Performed the experiments; Analyzed and interpreted the data; Contributed reagents, materials, analysis tools or data; Wrote the paper.

Bin Zong and Yi Liu: Performed the experiments; Analyzed and interpreted the data.

Mei Chen and Shanshan Li: Performed the experiments.

Dujuan Xu: Performed the experiments; Analyzed and interpreted the data; Contributed reagents, materials, analysis tools or data.

Bing Han: Conceived and designed the experiments.

### Data availability statement

Data will be made available on request.

### Additional information

No additional information is available for this paper.

## Declaration of competing interest

The authors declare that they have no known competing financial interests or personal relationships that could have appeared to influence the work reported in this paper.
